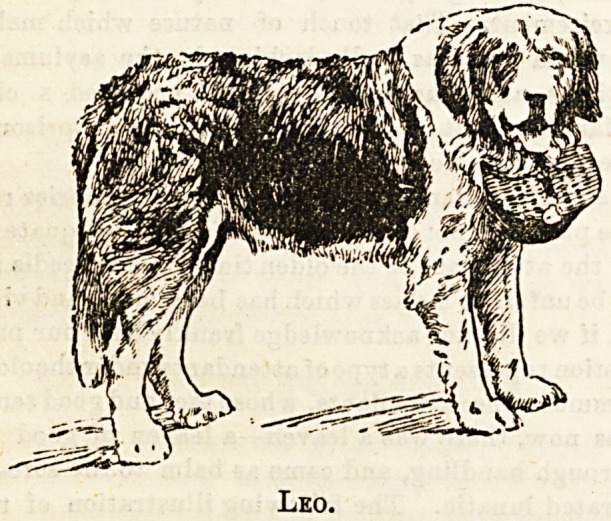# Around the Hospitals

**Published:** 1892-11-19

**Authors:** 


### Around the Hospitals.

The **Bristol General Hospital** has had time enough to prove the benefit of the new wards added to the institution since last year. The verdict is very satisfactory. The in- and out-patients treated during the year show a marked increase over those of the previous year; so that the work of the hospital has increased considerably. The extra expenditure for larger numbers has not been excessive. The subscriptions show but a slight advance, whilst the donations have been doubled this year. Legacies and the Sunday collections have realized less than formerly.

**The Children's Hospital, Cork.**—The canine friend, Leo, tells his own story: "I was a very young dog when I was first brought to the Children's Hospital in Cork, and introduced as a playmate to the little sick children. My dog language is quite inadequate to depict the sufferings I have witnessed in my short life. I am only three years old, yet it seems as if I had lived a lifetime. My heart has ached when I have lain in the hall and heard the little sick ones refused admittance for want of funds. Visitors used to pat me, praise my beauty, and say what a handsome fellow I was, and I began to think, Could I turn the gifts which nature had bestowed on me to account on behalf of the little sufferers? I was sent to the show, attained the status of a prize dog, got a hamper, put my prize money into it, and started collecting for the purpose of supporting a cot. I went

Leo.

round the show with my hamper, also attended an entertainment of 'Magpie Minstrels.' As I came in from the latter I found a poor widow in the hall in great distress, begging admission for her little lame child, who was with her. There was no free cot available at the time, and what was my delight when, on my hamper being emptied, and the money counted, to find I had enough in it to support a cot for the year, to be called after myself, 'The Leo Cot.' I watched this case with great interest, and at the end of three months my little patient was sent home to her mother quite well, and I overheard the doctors say that had not my little one been attended to in time she would have turned out a hopeless invalid. I am a very sagacious dog. Although I cannot say much I can understand what I hear, and, amongst other things, I heard some very painful facts regarding the funds of this hospital. I found out that it had no endowment of any kind, and was dependent on a very inadequate supply of voluntary contributions from year to year. The many readers of The Hospital have no doubt heard of the distressed state of poor Ireland, of the congested districts of poor Ireland, &c., but have they ever heard of the really distressed sick Irish children? I have made up my mind not to rest until I have endowed my cot, and for this purpose I make an urgent appeal to every reader of The Hospital to help me by sending donations, however small, even if only a few pence, addressed to 'Leo,' care of the Hon. Secretary, Women's and Children's Hospital, Infirmary Road, Cork, who will take care of the money for me, and see that it is safely banked to the Leo Account until the amount is realized—£300 is required. Don't refuse the dog's appeal. If every reader of this widely-circulated journal will send me a mite for my hamper my object will soon be attained."

## Figures and Tables

**Figure f1:**